# A Novel Imidazopyridine Derivative Exerts Anticancer Activity by Inducing Mitochondrial Pathway-Mediated Apoptosis

**DOI:** 10.1155/2020/4929053

**Published:** 2020-08-25

**Authors:** Juanli Wang, Hong Wu, Guiting Song, Donglin Yang, Jiuhong Huang, Xiaofang Yao, Hongxia Qin, ZhongZhu Chen, Zhigang Xu, Chuan Xu

**Affiliations:** ^1^National & Local Joint Engineering Research Center of Targeted and Innovative Therapeutics, Chongqing Engineering Laboratory of Targeted and Innovative Therapeutics, Chongqing Key Laboratory of Kinase Modulators as Innovative Medicine, Chongqing Collaborative Innovation Center of Targeted and Innovative Therapeutics, College of Pharmacy & International Academy of Targeted Therapeutics and Innovation, Chongqing University of Arts and Sciences, Chongqing 402160, China; ^2^Integrative Cancer Center & Cancer Clinical Research Center, Sichuan Cancer Hospital & Institute Sichuan Cancer Center, School of Medicine University of Electronic Science and Technology of China, Chengdu 610041, China

## Abstract

**Background:**

Cancer remains a major clinical challenge because of the lack of effective drug for its treatment. To find out novel cancer chemotherapeutic molecules, we explored the anticancer effect of novel imidazopyridine compound **9i** and also investigated the underlying molecular mechanism.

**Methods:**

Human cervical cancer cell (HeLa) viability was measured by an MTT assay after treatment with compound **9i**. Clonogenicity of HeLa cells was investigated by an in vitro colony formation assay. Cell death was visualized by propidium iodide (PI) staining. Fluorescence-activated cell sorting (FACS) was used to determine apoptosis and mitochondrial membrane potential in HeLa cells. The expression level of apoptosis-related proteins was also determined by western blot.

**Results:**

Compound **9i** suppressed HeLa cell viability in a time- and dose-dependent manner. Compound **9i** induced mitochondrial outer membrane permeabilization (MOMP), activated caspase cascade, and finally resulted in apoptosis.

**Conclusion:**

Compound **9i** induces mitochondrial pathway-mediated apoptosis in human cervical cancer cells, suggesting that **9i** could be a potential lead compound to be developed as a cancer therapeutic molecule.

## 1. Introduction

Imidazopyridines, one type of nitrogenous heterocycles, are largely used in medicinal application as they possess a wide spectrum of biological activities [1–3]. Imidazopyridines were reported to have anticancer properties in human cancer cell lines U251 (glioma), PC-3 (prostate), K-562 (leukemia), HCT-15 (colon), MCF7 (breast), SK-LU-1 (lung) [4], and LN-405 (glioblastoma) [5]. Based on these prominent anticancer effects of imidazopyridine compounds, we synthesized a series of imidazopyridine derivatives and tested their anticancer activity in various tumor cell lines [6]. In these compounds, compound **9i** (4-(8-bromo-3-(3-oxo-3,4-dihydroquinoxalin-2-yl) imidazo [1,2-a] pyridin-2-yl) benzonitrile) had the best anticancer effect on the human cervical cancer cell line HeLa. However, the underlying molecular mechanisms of compound **9i** inducing anticancer activity are completely unknown.

Apoptosis is programed cell death which is divided into two major subtypes, extrinsic and intrinsic apoptosis. Intrinsic apoptosis is initiated by mitochondrial outer membrane permeabilization (MOMP) [7]. Mitochondria are cellular energy generators, which are vital organelles in all kinds of eukaryotic cells [8]. At normal condition, mitochondria generate ATP to supply energy for intracellular metabolic pathways [9]. Upon a cytotoxic stimulus, mitochondrial outer membrane integrity was damaged (also known as MOMP) which results in cytochrome c release into the cytosol. The release of cytochrome c activates caspase-9 which activates effector caspases (caspase-3, caspase-6, and caspase-7), finally resulting in apoptosis [10]. MOMP is enhanced by proapoptotic proteins Bcl-2-associated X (BAX) and Bcl-2 antagonist killer (BAK); meanwhile, it is suppressed by antiapoptotic Bcl-2 family proteins (Bcl-2, Bcl-XL) [11, 12].

Here in this study, we further explored the molecular mechanism of compound **9i** inducing apoptosis in the human cervical cancer cell line HeLa. We found that compound **9i** decreased HeLa cell viability by inducing apoptosis. The following experiments revealed that **9i** upregulated proapoptotic proteins, induced MOMP, activated caspase cascade, and finally resulted in apoptosis. Taken together, the current research demonstrates that novel imidazopyridine compound **9i** induces mitochondrial pathway-mediated apoptosis in HeLa cells, which is the major incentive for its anticancer effect on cervical cancer cells. It suggests the potential role of **9i** as a promising novel anticancer agent.

## 2. Materials and Methods

### 2.1. Chemicals

Small molecular compound **9i** was synthesized by the chemical synthesis lab of IATTI as previously described [6]. Other regular chemical reagents were purchased from Sigma-Aldrich.

### 2.2. Cell Culture and Compound Treatment

The human cervical cancer cell line HeLa and noncancerous cell lines HEK293T and PNT1A used in this study were obtained from Cobier Biotechnology (Cobier, Nanjing, China). HeLa cells and PNT1A cells were maintained in RPMI 1640 medium (HyClone, SH30809.01, USA), HEK293T cells were maintained in DMEM (HyClone, SH30022.01, USA), with 10% fetal bovine serum (FBS, Gibco, 10100147, Australia) and 1% penicillin-streptomycin (Gibco, 15140122, Australia) added, and the cells were kept in a humidified atmosphere of 5% CO_2_ at 37°C. Compound **9i** was synthesized in our lab as previously described. Stock solution of compound **9i** was prepared in dimethyl sulfoxide (DMSO) and diluted in culture medium whenever needed. The final concentration of DMSO was less than 0.5% which did not affect cell survival.

### 2.3. Cell Viability Assay

The HeLa cells were cultured in 96-well plates with 3 × 10^3^ cells seeded. After 16-hour incubation, the medium was removed and 200 *μ*L fresh medium containing the indicated concentrations of compound was added. The compound-treated cells were cultured for another 24 hours, 48 hours, and 72 hours as indicated in the figure. The viability of cells was measured by an MTT assay based on the previous description [13]. 20 *μ*L of MTT solution (5 mg/mL in PBS) was added to each well and then incubated for another 4 hours. The medium was removed, and 150 *μ*L of DMSO was added to dissolve the formazan crystals. The optical density (OD) was measured with a microplate reader (BioTek, Winooski, VT, USA) at an absorbance wavelength of 570 nm. The survival rate of the compound-treated cells was compared to the equal amount of DMSO-treated cells. The IC_50_ of compound **9i** was measured after 48 hours of compound treatment.

### 2.4. Colony Formation Assay

The colony formation assay was based on the previous description [14]. The cervical cancer cell HeLa was seeded onto 6-well plates at a density of 500 cells per well containing 2 mL medium. After 16-hour incubation, cells were treated with compound **9i** at concentrations of 0, 5, 10, and 20 *μ*M for 48 hours. Cells were cultured in complete medium without **9i** for another 14 days, and cell culture medium was changed with fresh medium every 2 days. The cells were washed twice with PBS and fixed with 4% paraformaldehyde for 30 minutes, followed by staining with 0.5% crystal violet (Beyotime, C0121, Shanghai, China). Colonies were documented with an Epson scanner.

### 2.5. PI Staining Assay

HeLa cells (1 × 10^5^ cells/well) were seeded into 6-well plates. After 16 hours of attachment, the cells were treated with 5 *μ*M compound **9i** or DMSO for 24 hours. The cells were washed twice with PBS, followed by staining with PI (0.1 *μ*g/mL) and DAPI (0.5 *μ*g/mL) for 15 minutes at room temperature in the dark [15]. The images were obtained using an inverted fluorescence microscope (Olympus, Tokyo, Japan).

### 2.6. Apoptosis Assay

Cell apoptosis assays were based on the previous description [16]. Flow cytometry was performed with an annexin V-FITC apoptosis detection kit (Beyotime, C1063, Shanghai, China) according to the manufacturer's protocol. Cells were stained with propidium iodide (PI)/annexin V-FITC (annexin V-fluorescein isothiocyanate) and analyzed by flow cytometry. HeLa cells were treated with different concentrations of **9i** for 48 hours. All cells were harvested and washed twice with cold PBS, followed by incubation with PI and annexin V-FITC for 15 minutes at room temperature in the dark. The cell apoptosis was assessed by flow cytometry (Becton Dickinson, Accuri™ C6, USA).

### 2.7. Mitochondrial Membrane Potential (*∆*Ψm) Assay

The mitochondrial membrane potential was detected with rhodamine 123 (Sigma-Aldrich, USA) which is a *∆*Ψm-specific fluorescent probe. The experiments were performed as previously described [17]. Briefly, HeLa cells were treated with compound **9i** for 24 hours at different concentrations (0, 5, 10, and 20 *μ*M). The cells were washed twice with PBS, followed by incubation with rhodamine 123 and PI for 30 minutes at 37°C in the dark. The fluorescence intensities were analyzed using flow cytometry (Becton Dickinson, Accuri™ C6, USA) with the setting of FL1A at 530 nm and FL2H at 585 nm.

### 2.8. Western Blotting Analysis

The cultured HeLa cells were treated with compound **9i** or DMSO for 12 hours and lysed on ice for 30 minutes in radioimmunoprecipitation (RIPA) buffer (Beyotime, Shanghai, China) which contains a protease inhibitor and a phosphatase inhibitor (Roche, Mannheim, Germany). Fifty micrograms of total proteins was separated on 6~12% sodium dodecyl sulfate polyacrylamide gel electrophoresis (SDS-PAGE) and transferred onto a polyvinylidene difluoride (PVDF) membrane (Millipore Corporation, MA, USA). The membranes were blocked with 5% bovine serum albumin (Beyotime, ST023, Shanghai, China) in Tris-buffered saline containing 0.1% Tween 20 (TBST) for 1 h at room temperature and then incubated with primary antibodies (diluted to 1 : 1000, CST, USA) overnight at 4°C. The membranes were then incubated with horseradish peroxidase- (HRP-) labeled secondary antibodies (diluted to 1 : 10000, Sigma, USA) for 1 hour at room temperature. The immunoreactive bands were visualized with the ECL western blot detection kit (GE Healthcare, RPN3244, USA) on a Tanon 5200 Imaging System (Tanon Science & Technology Co., Ltd., Shanghai, China). The antibodies used in this research were as follows: caspase-9 (9502S), cleaved caspase-9 (9505S), caspase-3 (9662S), cleaved caspase-3 (9661S), tubulin (2128L), Bax (2774S), Bak (12105S), Bad (9239S), p-Bad (5284S), Bim (2933S), Bcl-2 (2872S), and Bcl-xL (2764S) (all from Cell Signaling Technology (CST), USA). The intensity of the immunoblotting bands was measured with ImageJ.

### 2.9. Statistics

All data were presented as mean ± SD from three independent experiments. All statistical analyses were carried out using a GraphPad Prism 5 software program for Windows (GraphPad Software, Inc.). Statistical significance was determined by Student's *t*-test (^∗^*p* < 0.05, ^∗∗^*p* < 0.01).

## 3. Results and Discussion

### 3.1. Compound **9i** Inhibits Cervical Cancer Cell Viability in a Time- and Concentration-Dependent Manner

**Figure 1:**
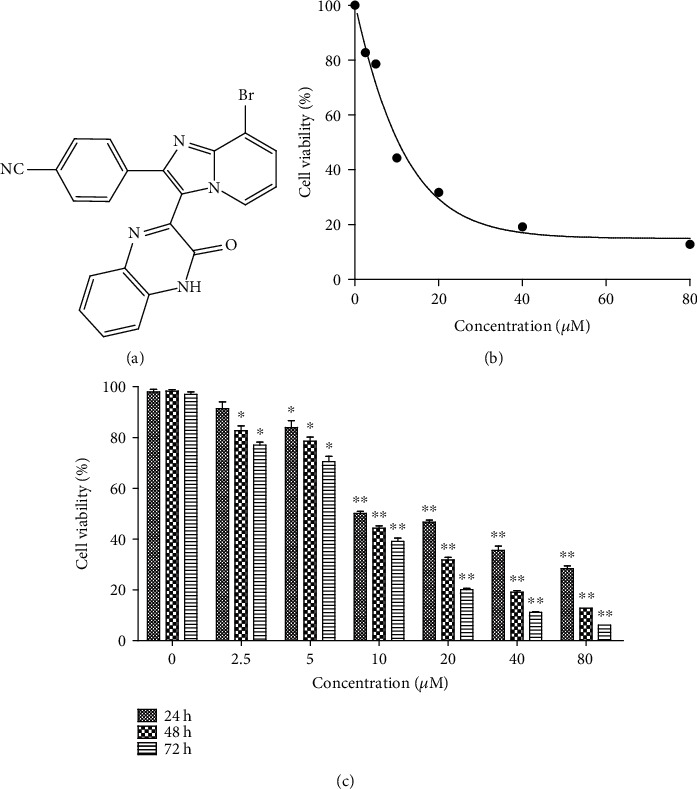
Compound **9i** inhibits HeLa cell survival in a time- and concentration-dependent manner. (a) Scheme of compound **9i**. (b) Hela cell viability after different concentrations of compound **9i** treated for 48 hours. (c) HeLa cell survival rate after indicated concentrations of compound **9i** treatment for 24, 48, and 72 hours, respectively. Cell viability was measured using the MTT assay. Survival rate equals to compound **9i**-treated cells compared to vehicle control cells; DMSO was used as a vehicle control. Data is represented as mean ± SD (*n* = 3). Significance was tested by Student's *t*-test (^∗^*p* < 0.05, ^∗∗^*p* < 0.01).

Our previous study found that a series of novel imidazopyridine analogues have anticancer activity in various tumor cell lines [6]. We want to further explore the molecular mechanism of the anticancer effect of these compounds. Compound **9i** (Figure 1(a)) was chosen for further research as it has the best anticancer effect.

To evaluate the anticancer effect of compound **9i**, cell viability was measured after compound **9i** treatment in human cervical cancer HeLa cell lines. The half maximal inhibitory concentration (IC_50_) of compound **9i** after 48-hour intervals was 10.62 *μ*M in HeLa cells (Figure 1(b)). To explore the dosage and temporal effect of compound **9i** on cancer cell survival, HeLa cells were treated with compound **9i** at different concentrations (0, 2.5, 5, 10, 20, 40, and 80 *μ*M) for 24, 48, and 72 hours, respectively, and the cell survival rate was measured. Cell viability decreases as the concentration of compound **9i** increased after 24-hour, 48-hour, and 72-hour intervals, respectively. The cell viability also decreases as the treated time increases under each concentration of compound **9i** treatment (Figure 1(c)). Compound **9i** did not affect normal human cell viability in human embryonic kidney 293T (HEK293T) cells and normal prostate epithelium PNT1A cells (Figure S1). The results indicate that **9i** decreases the HeLa cell survival rate in a time- and concentration-dependent manner.

### 3.2. Compound **9i** Inhibits Colony Growth

**Figure 2:**
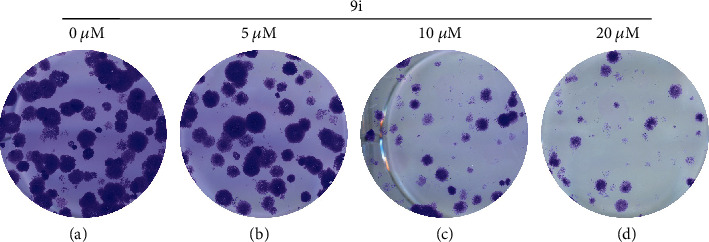
Compound **9i** inhibits colony growth. (a-d) Crystal violet staining of the HeLa cell colony after various dosages of compound **9i** (0, 5, 10, and 20 *μ*M) treatment. DMSO was used as a vehicle control.

To explore the effect of compound **9i** on clonogenicity, we conducted an in vitro colony formation assay. HeLa cells were treated with different concentrations of compound **9i** for 48 hours and cultured in fresh medium for another 14 days to let the colony grow. Colony size and numbers were dramatically decreased as the concentration of compound **9i** increased (Figure 2). The results indicate that compound **9i** inhibits human cervical cancer colony growth.

### 3.3. Compound **9i** Induces Cell Death

**Figure 3:**
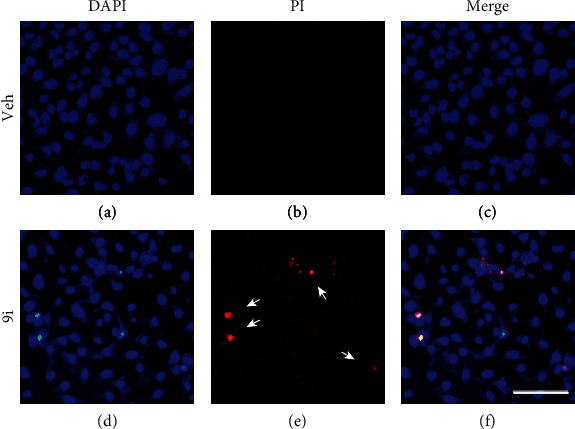
Compound **9i** induces cell death. Fluorescence images of (a, d) DAPI- and (b, e) PI-stained cells. HeLa cells were treated with (a–c) a vehicle control or (d–f) 5 *μ*M of **9i** for 24 hours and stained with DAPI and PI. Arrowheads indicate the cells with condensed chromatin and PI staining. DMSO was used as a vehicle control. DAPI was used to stain the nucleus. Scale bar represents 100 *μ*m.

The above results indicated that compound **9i** inhibited tumor cell viability; we want to know whether **9i** induces cell death. Propidium iodide (PI) is a small fluorescent molecule that binds to DNA. PI cannot pass through the cell membrane when the plasma membrane is intact. When the cell is undergoing cell death, the cell membrane becomes permeable which makes it possible for PI to traverse into the nucleus. So the dead cell can be stained and marked by PI. HeLa cells were treated with compound **9i** for 24 hours and stained with PI, compared with control (Figure 3(a)), compound **9i** treatment slightly decreased the cell number (Figure 3(d)). Part of the cells engulfed PI and emitted red fluorescence (Figure 3(e)), while the vehicle control cells were not stained with PI (Figure 3(b)). The results indicated that **9i** induces cell death in HeLa cells.

### 3.4. Compound **9i** Induces Apoptosis in a Concentration-Dependent Manner

**Figure 4:**
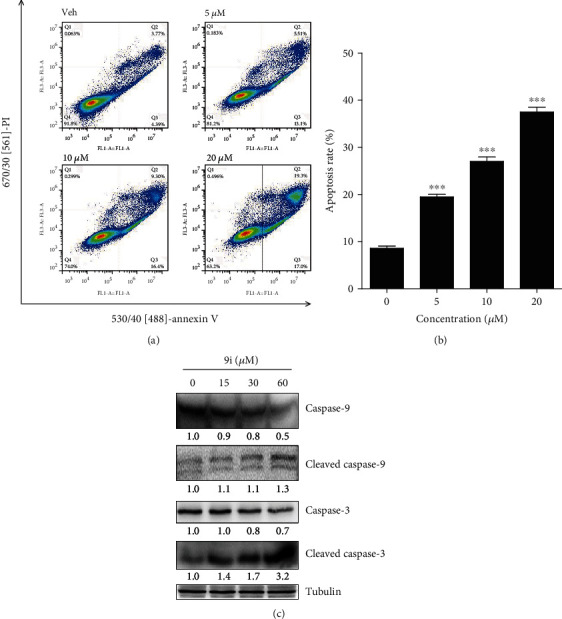
Compound **9i** induces apoptosis in human cervical cancer cells. (a) Representative scatter plots showing the distribution of annexin V and PI staining for control- and compound **9i**-treated HeLa cells. DMSO was used as a vehicle control. (b) Quantitative analysis of the percentage of apoptotic cells by FACS analysis. Values are represented as the mean ± SD (*n* = 3). Significance was tested by Student's *t*-test (^∗^*p* < 0.05, ^∗∗^*p* < 0.01 versus DMSO-treated cells). (c) Western blot was used to determine the protein level of caspase in DMSO- or compound **9i**-treated HeLa cells. The intensity of the bands was measured, and the fold change of the intensity was compared with that of the control and indicated below the bands. The expression of cleaved caspase-9 was statistically analyzed in Figure S2a. Tubulin was used as a loading control. The results were representative of three independent experiments.

We noted that in the dead cell stained with PI, the chromatin condensed to form a bright blue particle (Figures 3(d) and 3(f)), which is a remarkable process of apoptosis. The vehicle-treated HeLa cells showed no condensation or PI staining (Figures 3(a) and 3(c)). To validate whether compound **9i** induces apoptosis, we conducted fluorescence-activated cell sorting (FACS) to measure the apoptotic cells after annexin V-FITC/PI double staining. We found that as the concentration of compound **9i** increased, the apoptosis rate of the HeLa cells increased (Figures 4(a) and 4(b)).

Apoptosis is programed cell death regulated by cysteine aspartate protease (caspase) family proteins. We measured the protein levels of caspases and their activation forms in compound **9i**-treated HeLa cells by western blot. The protein level of full-length caspase-3 decreased, while its activation from cleaved caspase-3 significantly increased after **9i** treatment (Figure 4(c)), implying that caspase-3 is cleaved and activated by initiator caspase-9. Consistently, we found that the total level of caspase-9 decreased and cleaved caspase-9 increased as the concentration of **9i** increased (Figure 4(c)). The results indicate that compound **9i** activates caspase cascade and finally results in apoptosis.

### 3.5. Compound **9i** Induces Mitochondrial Pathway-Mediated Apoptosis

**Figure 5:**
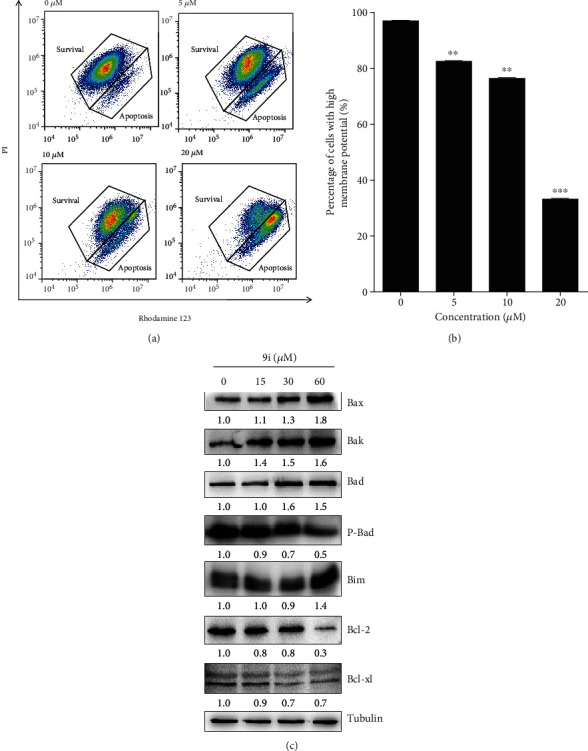
Compound **9i** induces mitochondria-dependent apoptosis in human cervical cancer cells. (a) Representative scatter plots showing the distribution of rhodamine 123 and PI staining for control- and compound **9i**-treated HeLa cells. DMSO was used as a vehicle control. (b) Quantitative analysis of the percentage of cells with high *∆*Ψm by FACS analysis. Values are represented as the mean ± SD (*n* = 3). Significance was tested by Student's *t*-test (^∗∗^*p* < 0.01, ^∗∗∗^*p* < 0.01 versus DMSO-treated cells). (c) Western blot was used to determine the expression level of apoptosis-related proteins in DMSO- or compound **9i**-treated HeLa cells. The intensity of the bands was measured, and the fold change of the intensity was compared with that of the control and indicated below the bands. The expression of Bcl-xL was statistically analyzed in Figure S2b. Tubulin was used as a loading control. The results were representative of three independent experiments.

Apoptosis is caused by a lot of pathways in cells. To clarify which pathway is involved in compound **9i**-induced apoptosis, we first checked the mitochondrial pathway activity after **9i** treatment. We examined whether the mitochondrial membrane integrity was damaged by compound **9i** treatment, and rhodamine 123 was employed to measure the mitochondrial membrane potential (*∆*Ψm) in the compound **9i**-treated Hela cells [18]. The results reveal that the cells with high *∆*Ψm decrease significantly as the concentration of **9i** increased (Figures 5(a) and 5(b)). It means that the mitochondrial pathway is activated in compound **9i**-induced apoptosis.

There are a lot of proteins involved in mitochondrial pathway apoptosis, and to determine whether compound **9i**-induced apoptosis is associated with B-cell/lymphoma 2 (Bcl-2) family proteins, the expression of proapoptotic (Bax, Bak, Bad, and Bim) and antiapoptotic (Bcl-2 and Bcl-xL) proteins was measured by western blot. The expression of proapoptotic proteins was significantly increased as compound **9i** concentration increased, while the expression of antiapoptotic proteins Bcl-2 and Bcl-xL was decreased (Figure 5(c)). These results reveal that compound **9i** triggers *∆*Ψm collapse, proapoptotic protein activation, and caspase protein activation and finally results in apoptosis. Taken together, compound **9i** induces mitochondrial pathway-mediated apoptosis.

Cervical cancer is a huge threat to women, while there is a great shortage of chemotherapeutic molecules for advanced cervical cancer [19]. Imidazopyridines are one type of nitrogenous heterocycles which have the ability to influence many cellular pathways for proper functioning of cancer cells, immune system, and enzymes involved in carbohydrate metabolism, etc. [20]. The imidazopyridine analogue O4I3 inhibits histone lysine demethylase 5 (KDM5) activity to promote reprogramming of resistant fibroblasts [21]. Numerous imidazopyridines have a prominent anticancer property in several human cancer cell lines [4, 5, 22]. Here in the current study, we explored the anticancer effect of novel imidazopyridine compound **9i** on the human cervical cancer cell line HeLa. The MTT assay showed that compound **9i** suppressed HeLa cell growth in a concentration- and time-dependent manner. Compound **9i** inhibited HeLa cell growth with IC_50_ of 10.62 *μ*M. Compound **9i** also inhibited HeLa cells' clonogenicity. All these data indicate that compound **9i** exhibits prominent anticancer properties in human cervical cancer.

To uncover the molecular mechanism of compound **9i** inhibiting HeLa cell growth, we checked the cell death of HeLa cells by PI staining. The results showed that HeLa cells were undergoing cell death, which were stained with PI after compound **9i** treatment. In addition, the PI-positive cells' chromatin condensed which is a characteristic phenomenon of apoptosis. Apoptosis is the most common mechanism in anticancer drug-induced cell death. The compound **9i**-induced apoptosis was demonstrated by FACS with PI/annexin V staining and western blot of caspase proteins. To dissect which pathway was involved in compound **9i**-induced apoptosis, the mitochondrial membrane potential was measured by FACS. The results indicated that compound **9i** induced MOMP in HeLa cells. Proapoptotic protein and antiapoptotic protein expression was measured by western blot, and compound **9i** induced downregulation of antiapoptotic proteins and upregulation of proapoptotic proteins. Taken together, compound **9i** induced mitochondrial pathway-mediated apoptosis in HeLa cells.

Our study revealed that compound **9i** was a cytotoxic reagent in HeLa cells and induced mitochondrial pathway-mediated apoptosis. However, these results were in cultured human cervical cancer cells and should be further confirmed in vivo, such as xenograft human tumor in mice. Since a cytotoxic reagent is a strategy to treat cervical cancer and many clinical trials are still undergoing, compound **9i** deserves further investigation to implement its anticervical cancer potential.

Some imidazopyridine derivatives have selectivity to many therapeutic targets for cancer therapy [23]. Two 2,6-disubstituted imidazo [4,5-b] pyridine compounds have high activity and selectivity against receptor tyrosine kinases AXL and MER in vitro [24]. Two new bisindole-imidazopyridine hybrids induce apoptosis in a human lung cancer cell line (A549) [25]. A new type of imidazopyridine compound selectively inhibits mTOR and exerts a strong antiproliferative effect against HeLa and NCM460 cell lines [26]. An apoptosis pathway is a typical pathway for targeted cancer therapy, a recent study found that Bcl-2 is a prominent target for cancer treatment [27], and many small molecules were designed to target Bcl-2 [28]. Our study found that compound **9i** significantly decreased Bcl-2 expression, and whether compound **9i** targeted Bcl-2 or other molecules in the apoptosis pathway needs further exploration.

## 4. Conclusions

To draw a conclusion, compound **9i** showed high cytotoxicity on cervical cancer cells. The molecular mechanism behind its cytotoxicity is that **9i** induces MOMP, activates caspase cascade, and finally results in apoptosis. Hence, compound **9i** could be further exploited as a potential lead compound in human cervical cancer therapy.

## Data Availability

No data was used to support this study.

## Supplementary Materials

Supplementary Materials Figure S1: compound **9i** did not affect normal human cells' viability. HEK293T (a) cell and PNT1A (b) cell viability was measured after different concentrations of compound **9i** treated for 48 hours. There was no significant difference between each concentration group. Figure S2: relative fold change in the protein expression of cleaved caspase-9 (a) and Bcl-xL (b) in western blot images from Figures 4 and 5. Significance was tested by Student's *t*-test (^∗^*p* < 0.05 versus DMSO-treated cells). The results were representative of four independent experiments.
