# Supplementary material for: A Novel Imidazopyridine Derivative Exerts Anticancer Activity by Inducing Mitochondrial Pathway-Mediated Apoptosis
Source: Biomed Res Int. 2020 Aug 25;2020:4929053. doi: 10.1155/2020/4929053 (PMC7468608; doi:10.1155/2020/4929053)
Supplement: Supplementary Materials — Figure S1: compound 9i did not affect normal human cells' viability. HEK293T (a) cell and PNT1A (b) cell viability was measured after different concentrations of compound 9i treated for 48 hours. There was no significant difference between each concentration group. Figure S2: relative fold change in the protein expression of cleaved caspase-9 (a) and Bcl-xL (b) in western blot images from Figures 4 and 5. Significance was tested by Student's t-test (∗p < 0.05 versus DMSO-treated cells). The results were representative of four independent experiments. [file 4929053.f1.pdf]

Supplemental figures

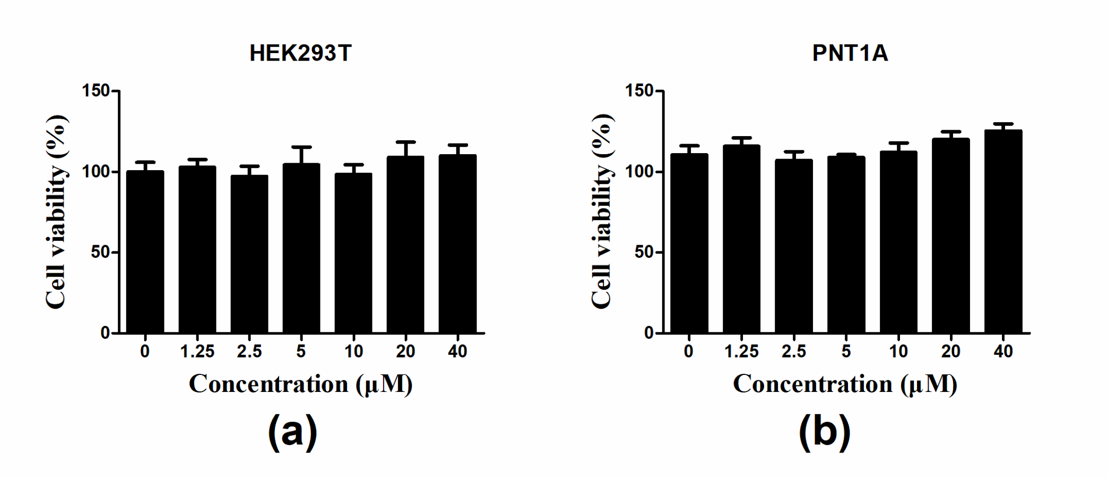

Figure S1. Compound **9i** did not affect normal human cells' viability. HEK293T (a) cells and PNT1A (b) cells viability was measured after different concentration of compound **9i** treated for 48 hours. There was no significant difference between each concentration group.

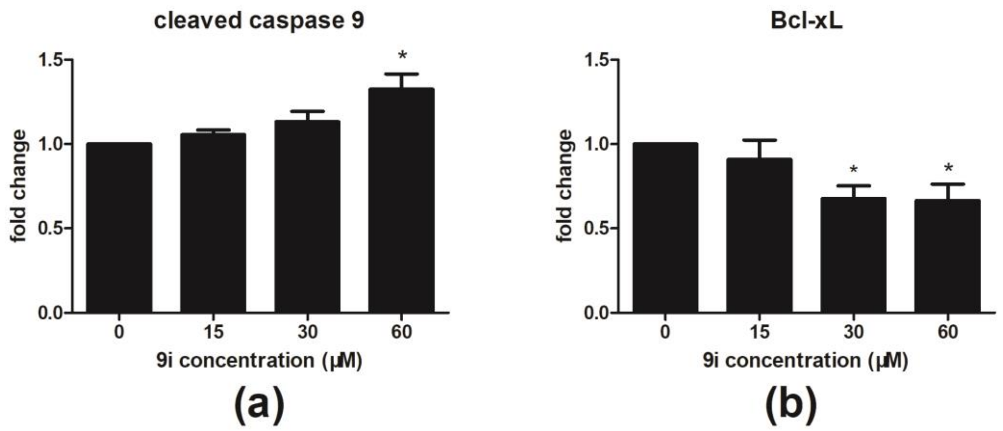

Figure S2. Relative fold change in protein expression of cleaved caspase 9 (a) and Bcl-xL (b) in western blot images from Figure 4 and Figure 5. Significance was tested by Student t-test; *: $p<0.05$ versus DMSO treated cells. The results were representative of four independent experiments.
